# Ab Initio Study of Electron Capture in Collisions of Protons with CO_2_ Molecules

**DOI:** 10.3390/molecules30010074

**Published:** 2024-12-28

**Authors:** Luis Méndez, Ismanuel Rabadán

**Affiliations:** Departamento de Química, Universidad Autónoma de Madrid, Cantoblanco, 28049 Madrid, Spain; luis.mendez@inv.uam.es

**Keywords:** ion–molecule collisions, charge transfer, configuration interaction calculations

## Abstract

Ab initio calculations of cross sections for electron capture by protons in collisions with CO_2_ are carried out at energies between 100 eV/u and 50 keV/u, employing a semiclassical method within the Franck–Condon framework. The scattering wave function is expanded in a set of ab initio electronic wave functions of the HCO_2_^+^ supermolecule. The calculation is performed on several trajectory orientations to obtain orientation-averaged total cross sections. A two-state model with an exponential interaction between the entrance and the lowest charge transfer channel is proposed to describe the main aspects of the charge transfer process and to estimate the precision of the molecular expansion. The symmetry of the HOMO $\pi_{g}$ of CO_2_ is relevant to choose the signs of the molecular functions and to set up the orientation average of the cross sections. Very good agreement is found with the experimental charge transfer cross sections.

## 1. Introduction

Carbon dioxide (CO_2_) is the most abundant molecule in some planetary atmospheres, like Venus and Mars, while its concentration on Earth has risen steadily during the last 300 years [1], contributing to the greenhouse effect and global warming scenarios. The interactions of solar wind particles with CO_2_ can produce its ionization and even fragmentation, so it is important to accurately determine the cross sections of these processes. While electron collisions with CO_2_ have been widely studied since 1920, and these works have been reviewed several times (see, for example, Itikawa [2], McConkey et al. [3]), studies of proton collisions with CO_2_ are very scarce. Experiments of charge transfer (CT) in H^+^ + CO_2_ collisions have been carried out in references [4,5,6,7,8,9,10]. Single ionization and charge exchange in CO_2_ by protons was also measured in Ref. [11] for impact energies above 50 keV/u, determining that molecular fragmentation is about 20% of the single ionization cross section at that energy. Moretto-Capelle et al. [12] measured the three-body fragmentation of CO_2_ in collisions with 25 keV protons, and Bhardwaj et al. [13] investigated the fragmentation of CO_2_^+^ ions in collisions with several neutral targets. In addition, the fragmentation of CO_2_ in collisions with He^+^ has been studied in reference [14]. On the theoretical side, we only find the calculations by Werbowy and Pranszke [10] using the ion–alkali atom model of Olson [15].

The aim of this paper is to study the charge exchange process in H^+^ + CO_2_ collisions for energies 0.1≤E≤50keV/u. In this energy range, we employ the semiclassical eikonal treatment, where the ion–molecule relative motion is described by means of classical trajectories while the electronic motion is described quantally. Since the experiments provide orientation-averaged cross sections (OAXS), we carry out calculations for several molecular orientations with respect to the ion beam (or, alternatively, several trajectory orientations with respect to the target axis) to obtain orientation-averaged results. The early work of Errea et al. [16] in H^+^ + H_2_ calculated the OAXS for charge transfer, using three trajectory orientations relative to a fixed molecule [17], and it showed that, for that system, this average leads to cross sections in good agreement with a simple isotropic approximation. In the latter calculation, the collision is treated as an ion–atom collision with the energies and dynamical couplings calculated assuming that the projectile approaches the molecule along trajectories with fixed values of the angle γ, with $\cos\gamma =\hat{R}\;\cdot \;\hat{\rho }$, where ρ is the H_2_ internuclear vector and ***R*** the position vector of the projectile with respect to the midpoint of the H–H internuclear axis. This is the average previously applied in the infinite order sudden approximation [18]. Furthermore, it was found [16] that, for such a simple target, a single calculation with γ in the interval $45^{\circ }$–$60^{\circ }$ provides a good approximation to the OAXS. In this work, we apply a more detailed average procedure, suitable for its application within the semiclassical formalism.

The calculation of OAXS within the semiclassical framework involves the previous calculation of electronic energies and non-adiabatic (also called dynamical) couplings along the projectile trajectory. This method, which uses ab initio electronic wave functions obtained along the trajectory, has been applied to study H^+^ + BeH collisions by Rabadán and Méndez [19] and to Sn^3+,2+^ + H_2_ collisions by Rai et al. [20] and Bijlsma et al. [21]. In the present paper, we extend the application of the method to ion collisions with a three-center target. An important distinction of the present study compared to previous works lies in the nature of the molecular orbital from which the exchanged electron is released. In this study, a large contribution to the CT cross section originates from the $\pi_{g}$ orbital of the CO_2_ molecule, whereas in previous studies, the electron was removed from a σ orbital. This difference in orbital symmetry is highly significant, as the $\pi_{g}$ orbital exhibits angular nodes that necessitate a more detailed treatment of molecular orientation during the collision. Also, achieving accurate orientation-averaged cross sections for the $\pi_{g}$ orbital demands the inclusion of a larger number of collision trajectories, reflecting the increased complexity introduced by the orbital’s anisotropic symmetry. This point is particularly relevant for extending the calculations to collisions with large molecules as in references [22,23].

The paper is organized as follows: We compare the calculated OAXSs with the available experimental results in Section 2. In Section 3, we present the method used, including the quantum chemistry calculation of the electronic states of the HCO_2_^+^ system, and the dynamical calculation. We outline the main conclusions of the work in Section 4. Atomic units are used unless otherwise stated.

## 2. Results and Discussion

### 2.1. Molecular Calculations

In the molecular method, the collision wave function is expanded in a set of electronic functions of the system HCO_2_^+^ (see Equation (11) in Section 3). In our model, the energies of the electronic states and the dynamical couplings (13) are calculated along ion trajectories with the CO_2_ molecule fixed. We have considered trajectory families with the ion velocity parallel to the CO_2_ axis, denoted as t_‖_, and with the velocity perpendicular to the molecular axis (t_⊥α_) as explained in detail in Section 3.1. The quantum chemistry method to obtain the molecular electronic wave functions is detailed in Section 3.2. The calculated energies of the channels, at the largest distances considered, are presented in Table 1, setting as zero energy that of the entrance channel H^+^ + CO_2_($X^{1}\Sigma_{g}^{+}$).

As specified in Table 1, the wave functions of A’ symmetry (C_*s*_) are expanded in about $3.3\times 10^{5}$ contracted CSFs, while those of A symmetry (C_1_) require about $8.0\times 10^{5}$ CSFs. The asymptotic energy difference between the entrance channel and the first CT channel is about −0.1 eV in our calculation, while the experimental adiabatic ionization energy of Wang et al. [24] leads to an energy difference of 0.17 eV. The calculated energy of the second CT channel is about 0.3 eV higher than the experimental value [25].

The entrance channel and the main exit CT channel are quasi-degenerate asymptotically, which in practice means that the order of the electronic energies of both states depends on the relative accuracy of the calculations. In our model, the energy of the entrance channel (H^+^ + CO_2_) is higher than that of the first exit channel, which disagrees with the experimental adiabatic ionization energy. To explain this situation, one has to take into account that, at large H-CO_2_ distances, the computational effort required to describe the electronic structure of the CO_2_^+^ ion is smaller than for the neutral CO_2_. Consequently, a systematic improvement of the CASSCF-MRCI calculation (see Section 3.2) does not necessarily lead to a better ionization energy. In the calculation, the CT reaction to the main exit channel is exothermic, which could artificially increase the CT cross section at low collision energies. The relevance of this effect in the CT calculation is tested by applying the two-state model outlined in Section 2.2.

**Figure 1:**
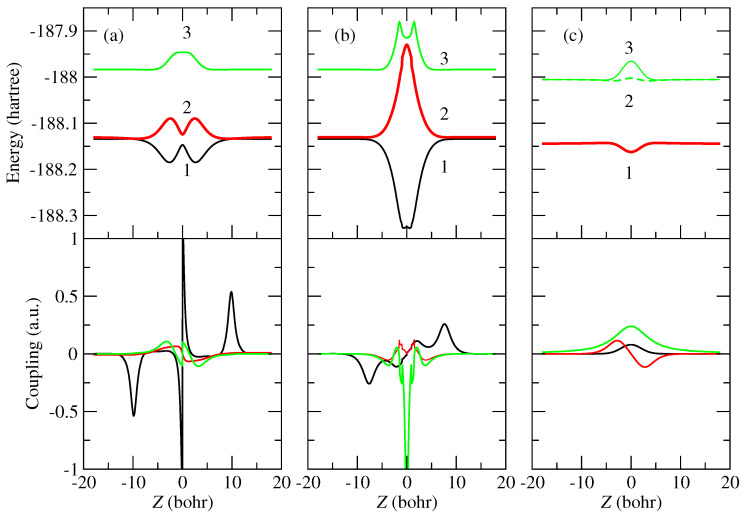
Potential energy curves (top panels) and dynamical couplings (bottom panels) along trajectories (**a**) t_‖_, (**b**) t_⊥0_, and (**c**) t_⊥90_, with b=4.0 bohr. The dynamical couplings shown in the bottom panels are coupling $M_{12}$, black lines; $M_{13}$, red lines; and $M_{23}$, green lines. The adiabatic states are numbered in increasing energy order, as shown in the top panels. The two green lines in the energies of panel (**c**) correspond to the two molecular states dissociating in H(1s) + CO_2_^+^ ($A^{2}\Pi_{u}$).

As an illustration of the molecular calculation, in Figure 1 we show the energies and dynamical couplings along the trajectories t_‖_, t_⊥0_ and t_⊥90_. Along the trajectory t_‖_, the system keeps C_*s*_ symmetry. The electronic states, which are antisymmetric with respect to reflection in the collision plane (b, v), are not coupled to the state dissociating into H^+^ + CO_2_($X^{1}\Sigma_{g}^{+}$) and, therefore, they are not populated during the collision. Accordingly, in Figure 1 we plot only the energies and couplings of the symmetric electronic states dissociating into H(1s) + CO_2_^+^($X^{2}\Pi_{g}$) and H(1s) + CO_2_^+^ ($A^{2}\Pi_{u}$). Analogously, in trajectory t_⊥0_, the antisymmetric wave functions with respect to the collision plane (b, v) are not coupled with the entrance channel and the corresponding electronic states are not populated during the collision.

For trajectories t_⊥90_, the symmetry under reflection on the (b, v) plane, which is perpendicular to the CO_2_ axis, leads to vanishing couplings with the states dissociating into H(1s) + CO_2_^+^ ($X^{2}\Pi_{g}$), and we only present the couplings with the molecular states dissociating into H(1s) + CO_2_^+^ ($A^{2}\Pi_{u}$). The relatively large energy difference between the entrance channel and the second CT channel yields a small transition probability, and we expect a small contribution of this trajectory orientation to the charge-transfer process.

In our calculation for trajectory t_‖_ (see Table 1), the electronic state labeled 1 in Figure 1a correlates to the symmetric component, under reflection on the (b, v) plane, of H(1s) + CO_2_^+^ ($X^{2}\Pi_{g}$). The adiabatic state labeled 2 dissociates into the entrance channel H^+^ + CO_2_($X^{1}\Sigma_{g}^{+}$), and the adiabatic state 3 correlates to the symmetric component of H(1s) + CO_2_^+^ ($A^{2}\Pi_{u}$). The long-range interactions are more intense for state 2 than for state 1, which leads to the avoided crossing between the potential energy curves at Z≈±10 bohr, which corresponds to the peaks in the $M_{12}$ coupling see Equation (13)). The $M_{12}$ coupling increases at low *Z* where the energy curves approach, and there is a sudden sign change at Z=0. The signs of the adiabatic states $\chi_{k}$ obtained in the MRCI procedure are undefined and, in practice, they are chosen to ensure that the time evolution of the adiabatic functions and, accordingly that of the dynamical couplings, is smooth. However, in this particular collision system, and due to the antisymmetric nature of the $\pi_{g}$ MO (molecular orbital) under reflection on the Z=0 plane, the gradual change in molecular functions results in a sign reversal in the $M_{12}$ coupling. The $M_{12}$ coupling also shows a sign flip in trajectory t_⊥0_, where the $\pi_{g}$ MO is also antisymmetric under reflection on the plane Z=0. We present a detailed explanation of this sign swapping in Appendix A.

For other trajectories perpendicular to the molecular axis, included in the averaging procedures (21)–(23), the symmetry restrictions mentioned above do not hold and we carry out 5-state dynamical calculations. Also, the sign change at Z=0 is smoother than for trajectories t_‖_ and t_⊥0_.

### 2.2. Two-State Model

**Figure 2:**
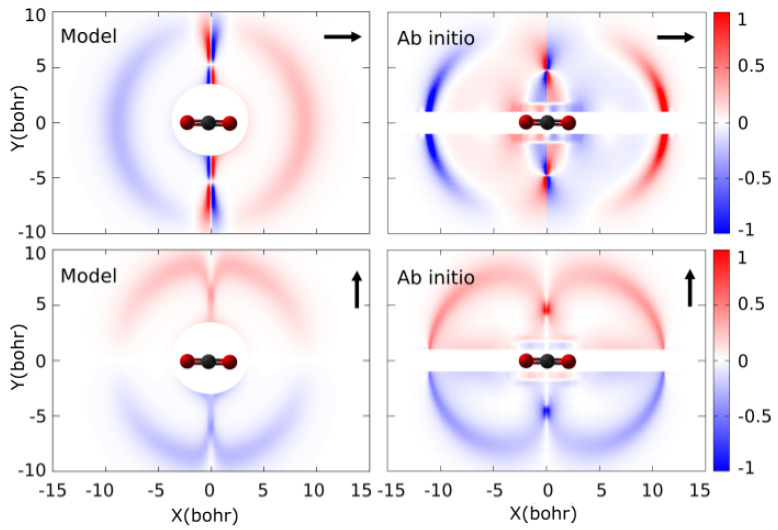
Color maps of the dynamical couplings, $\langle \chi_{1}\left|d/dZ\right|\chi_{2}\rangle$, between the first two electronic states of (H + CO_2_)^+^ system for trajectories parallel (top) and perpendicular (bottom) to the CO_2_ molecular axis. Comparison between the Demkov model (Equation (8)) with $E_{0}=0.006$ hartree and the ab initio data.

In this section, we introduce a simple two-state model, similar to the Demkov model [26], to describe the main features of the collision in trajectories parallel to the CO_2_ axis (t_‖_). An analogous model can be developed for other trajectories, and, in particular, in Figure 2 we also present the comparison with the ab initio calculations for t_⊥0_. The model involves the long-range energies of the entrance channel H^+^ + CO_2_($X^{1}\Sigma_{g}^{+}$) and the main CT channel H(1s) + CO_2_^+^($X^{2}\Pi_{g}$). In the Demkov model, the CT takes place by transitions between two diabatic states with constant energies ($H_{11}$, $H_{22}$) and an exponential interaction ($H_{12}$). The diagonalization of the Hamiltonian matrix leads to the adiabatic energies. To describe the dependence on the projectile-target distance and on the trajectory orientation, we have included long-range interactions in the diagonal matrix elements. In this respect, we used a related model (Langevin–Demkov) [27] for ion–atom collisions, where the diagonal matrix elements included the ion-induced dipole interaction, as in the Langevin model [28]. Explicitly:

(1) $$\begin{array}{ccc} H_{11}\left(b,Z\right) & = & E_{0}-\frac{p\left(Z\right)Q_{⊥}+q\left(Z\right)Q_{\|}}{2R^{3}}-\frac{p\left(Z\right)\alpha_{⊥}+q\left(z\right)\alpha_{\|}}{2R^{4}} \end{array}$$

(2) $$\begin{array}{ccc} H_{22}\left(b,Z\right) & = & -\frac{\alpha_{H}}{2R^{4}} \end{array}$$

where (b,Z=vt) are the proton coordinates with respect to the C nucleus and $R={\left(b^{2}+Z^{2}\right)}^{1/2}$. $E_{0}$ is the energy difference in the limit Z→∞, which is identical to the difference between the adiabatic energies in this limit. $\alpha_{⊥}=13.018$ a.u.^3^, $\alpha_{\|}=27.25$ a.u.^3^ are the polarizabilities of CO_2_ perpendicular and parallel to its axis [29], and $Q_{⊥}=1.59$ a.u.^2^, $Q_{\|}=-3.18$ a.u.^2^ [30] are the diagonal elements of the quadrupole tensor. The orientation of the internuclear vector ***R*** changes along the trajectory. Therefore, we have introduced, for Z>0, the switching function, p(Z):

(3) $$\begin{array}{ccc} p(Z) & = & \frac{2.0}{1.0+e^{-2.0|Z|}}-1.0 \end{array}$$

(4) $$\begin{array}{ccc} q(Z) & = & 1-p(Z), \end{array}$$

which connects the ion-quadrupole and ion-induced-dipole contributions to the energy of the entrance channel in the limits Z→0 and Z→∞. The interaction term has the form:

(5) $$H_{12}\left(b,Z\right)=a_{0}\;p\left(Z\right)\exp\left(-a_{1}R\right),$$

which includes two fitting parameters $a_{0}$, $a_{1}$. Inclusion of the switching function p(Z) removes the interaction in the limit Z→0 where the two wavefunctions have different symmetries with respect to the reflection in the XY plane. In this two-state model, the energy difference between the adiabatic states is related to the matrix elements $H_{ij}$ by

(6) $$\Delta E_{a}\left(Z,b\right)={\left[{\left(H_{22}-H_{11}\right)}^{2}+4H_{12}^{2}\right]}^{1/2}$$

and the values of $a_{0}=2.5$ and $a_{1}=0.85$ were determined by ensuring that the adiabatic energy difference closely matches the calculated value.

To express the dynamical coupling in the two-state model, we introduce the transformation angle:

(7) $$\Theta \left(Z,b\right)=\frac{1}{2}\arctan\left(\frac{2H_{12}}{H_{22}-H_{11}}\right)$$

and, for Z>0, the dynamic coupling between the adiabatic states is:

(8) $$\langle \chi_{1}\left|\frac{d}{dZ}\right|\chi_{2}\rangle =\frac{d\Theta }{dZ}.$$

The model does not include the translation factor corrections; see (15).

The diagonal elements $H_{11}$, $H_{22}$ are symmetric with respect to the sign change of *Z*, but the dynamical coupling has a sign flip at the point of closest approach that we have introduced ad hoc in the model by multiplying the result of (8) by sgn(Z). In Figure 2, we compare the couplings obtained in the model for t_‖_ and t_⊥0_ with the ab initio ones in the color plots of Figure 2. Taking into account that the model is based on the asymptotic behavior of the molecular wave functions, and the lack of translation factors, reasonable agreement is found with the ab initio couplings.

**Figure 3:**
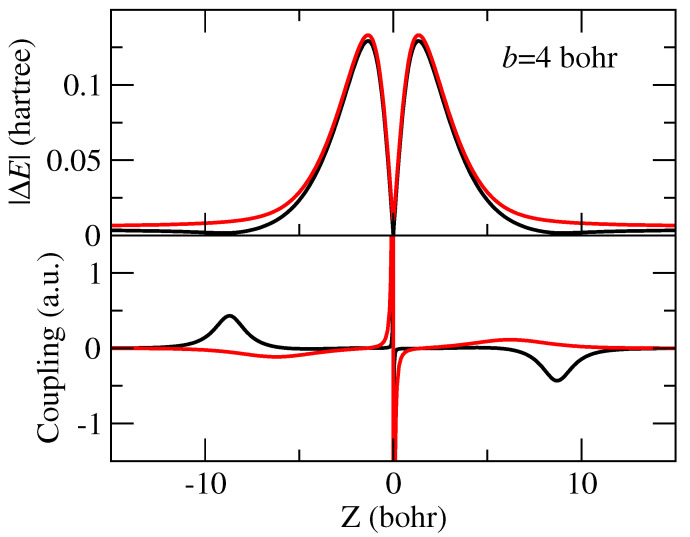
Energy difference and coupling used in the 2-state model of Section 2.2 with $E_{0}=+0.004$ hartree (black lines) and $E_{0}=-0.006$ hartree (red lines) to estimate the error produced by the swapping of asymptotic energies, while keeping the same interaction, on the CT cross-section.

In Figure 3, we compare the energies and the dynamical coupling for two values of the asymptotic energy difference $E_{0}$. The value $E_{0}=-0.006$ hartree (−0.16 eV) corresponds to the adiabatic experimental ionization energy of CO_2_ while $E_{0}=+0.004$ hartree (0.11 eV) is similar to the calculated energy difference. It can be noted that the outer peaks of the coupling appear for $E_{0}>0$ as a consequence of the avoided crossing, as already found in the ab initio calculation (Figure 1).

### 2.3. Dynamical Results

**Figure 4:**
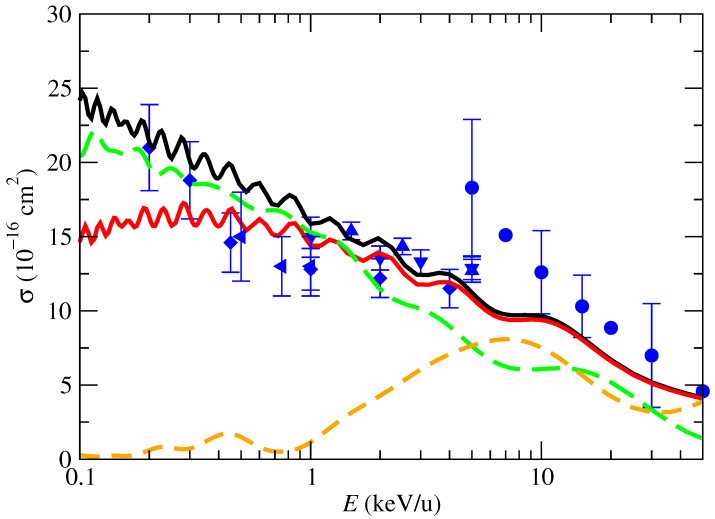
Charge transfer cross sections obtained with the two-state models and the ab initio results for trajectories parallel to the CO_2_ axis (t_‖_). The full lines are the results from the models with $E_{0}=+0.004$ hartree (black line) and $E_{0}=-0.006$ hartree of (red line). The dashed lines are the ab initio results for production of CO_2_^+^($X^{2}\Pi_{g}$) (long-dashed green) and for production of CO_2_^+^($A^{2}\Pi_{u}$) (short-dashed orange). Symbols are experimental data: • [5]; ▴ [6]; ⧫ [7]; ▾ [8]; ◂ [10].

In this section, we present the calculated CT cross sections. To check the precision of our results, in Figure 4 we compare the orientation-dependent CT cross section ${\hat{\sigma }}_{\mathrm{CT}}^{\|}$ (see the definitions in Section 3.1), calculated with the two-state model of Section 2.2 for trajectory t_‖_ and for the two values of $E_{0}$ considered in Figure 3. In this figure, we also include the partial cross sections to populate the CT states $X^{2}\Pi_{g}$ and $A^{2}\Pi_{u}$, obtained in the 3-state ab initio calculation, also for t_‖_. The cross section of the model calculation with $E_{0}=+0.004$ hartree agrees with that of the 3-state ab initio calculation for E≤2keV/u. At higher energies, the CT into $A^{2}\Pi_{u}$ starts to become sizable and the two-state model is less accurate.

The application of the model shows that the estimated overestimation of the calculated cross sections by effect of the swapping or the asymptotic energies is only noticeable at energies below 0.8 keV/u, and the differences between both results at E<0.8keV/u are of the order of 25%, while the uncertainties of the experimental data are ±17%. At E≥1keV/u, the estimated uncertainty of our calculation is lower than 10%, which supports our molecular expansion.

**Figure 5:**
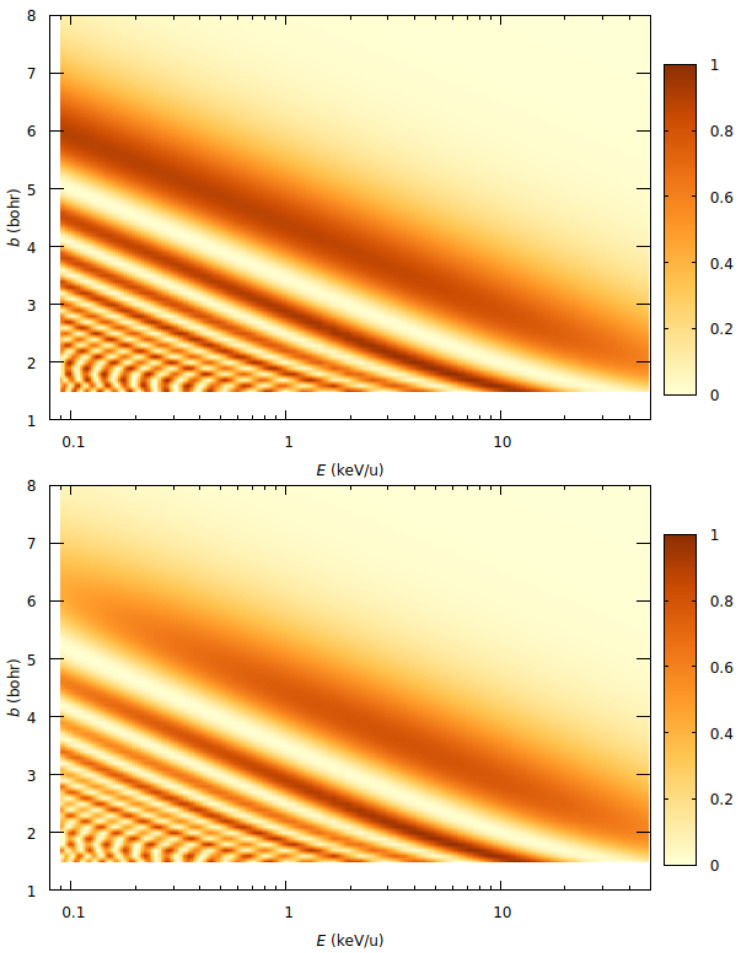
Color maps of the charge transfer probability in proton collisions with CO_2_ as a function of the impact parameter *b* and the impact energy *E* with the 2-state model of Section 2.2 with $E_{0}=-0.006$ hartree (top) and $E_{0}=+0.004$ hartree (bottom).

A more stringent test on the effect of a shift of the electronic energies is provided by the comparison of the corresponding transition probabilities (see Equation (16)). In the color maps of Figure 5, we show the probabilities ${\hat{P}}_{kl}^{\|}\left(b,v\right)$, calculated with the two-state model and two values of $E_{0}$: $E_{0}=-0.006$ hartree and $E_{0}=+0.004$ hartree. One can note the good agreement between the oscillatory structure of the probabilities from both calculations, except for the low-energy region, already shown in the cross sections of Figure 4, indicating that the agreement between the cross sections from both calculations is not coincidental.

**Figure 6:**
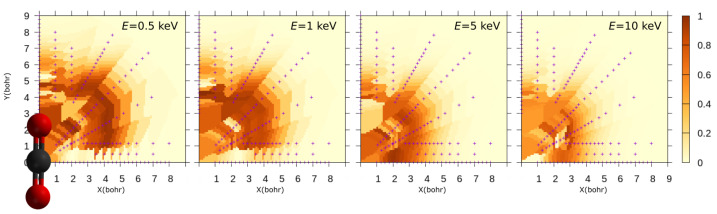
Color maps showing the probability of electron capture as function of the position of the impact parameter vector, b in the Cartesian plane, with ***v*** perpendicular to this plane. Each panel corresponds to a different impact energy, as labeled. The position of the molecule in the coordinate system is also shown.

To study the orientation dependence of our results, in Figure 6 we present the color map of the CT probability for the trajectory family perpendicular to the molecular axis of CO_2_, obtained through linear interpolation of the ab initio data ${\hat{P}}_{\mathrm{CT}}^{⊥}\left(b\right)$, indicated by points marked with a “+” symbol. Due to the symmetry of CO_2_, only one quadrant is shown. The maps correspond to collision energies of 0.5, 1, 5, and 10 keV/u. We find that at 0.5 keV/u and 1 keV/u, the trajectories with small “y” values exhibit very low probabilities. This region around the molecule corresponds to trajectories with α≈π/2 (see figure in Section 3.1), near the symmetry plane perpendicular to the molecular axis, where the HOMO orbital ($\pi_{g}$) presents a nodal plane that inhibits electron capture, as already found in the dynamical couplings. For these trajectories, the electron capture originates from deeper orbitals, beginning with HOMO-1 ($\pi_{u}$), which is active at collision energies of 5 and 10 keV, as shown in the figure. The probability maps also display isotropy, except for the aforementioned trajectories, with a maximum transition probability at impact parameters 4 bohr and 5 bohr for E=0.5keV/u, decreasing to around 3 bohr at E=10keV/u.

**Figure 7:**
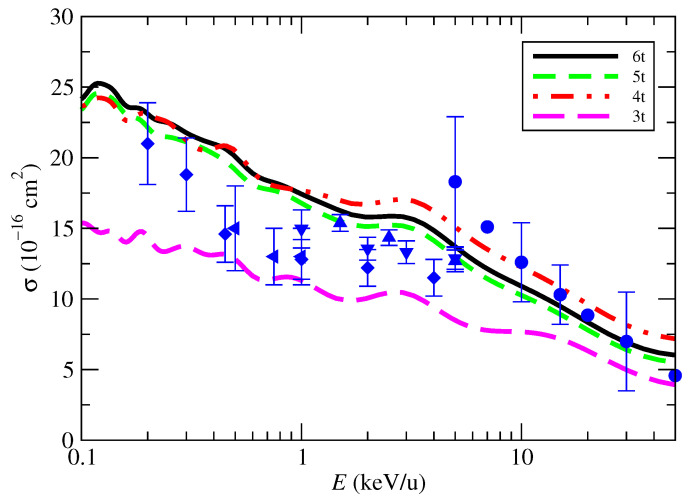
Orientation-averaged CT cross-sections in proton collisions with CO_2_ as functions of the impact energy. The lines are the present calculations of the total OAXS, obtained by averaging over different number of trajectory orientations: 3t, (20); 4t, (21); 5t, (22); and 6t, (23), as indicated in the figure. Experimental results: • [5]; ▴ [6]; ⧫ [7]; ▾ [8]; ◂ [10].

In Figure 7, we compare the OAXS calculated with the 3-, 4-, 5-, and 6-trajectory formulae of Equations (20)–(23). One can note that the OAXS derived from the 3t formula is noticeably lower than the other results and the experimental data. This discrepancy arises because the 3t formula includes a relatively significant contribution from the t_⊥90_ trajectory, which contributes nothing to the population of the primary exit channel, CO_2_^+^(X). This bias disappears when we include fewer symmetric trajectories, as in the other average models. The comparison also shows very good agreement between the 4-, 5-, and 6-trajectory results, which indicates that the process of incorporating more orientations of the impact parameter for the trajectory perpendicular to the internuclear axis has converged.

**Figure 8:**
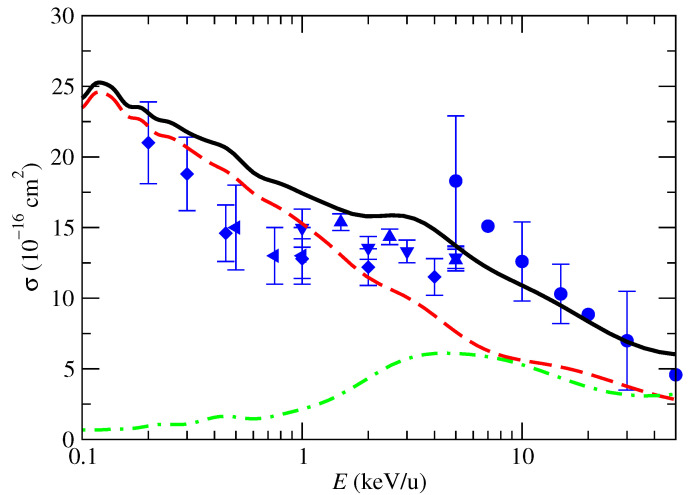
Orientation-averaged charge-transfer cross section in proton collisions with CO_2_ as a function of the impact energy. Lines are the present calculations: solid line, total charge transfer; red-dashed line, charge transfer to CO_2_^+^(X); and green dashed-dotted line, charge transfer to CO_2_^+^(A). Symbols are experimental data: • [5]; ▴ [6]; ⧫ [7]; ▾ [8]; ◂ [10].

The orientation-averaged partial cross sections for CT into CO_2_^+^(X) and CO_2_^+^(A) are presented in Figure 8. As already found for the cross sections ${\hat{\sigma }}_{\mathrm{CT}}^{\|}$ (Figure 4), the OAXS for CT into CO_2_^+^(A) is negligible for energies below 1 keV/u; it increases at higher energies and becomes competitive with the CT into CO_2_^+^(A) at E>5keV/u. There are not experimental data available of these partial cross sections. In the energy range of Figure 8, we find remarkably good agreement between the calculated total CT OAXS and the experimental data. The extension of the calculation to low energies, E<0.1keV/u, requires the description of the target vibrational motion and eventually quantal effects.

## 3. Materials and Methods

### 3.1. Semiclassical Treatment

At the relatively high collision energies of the present calculation, one can apply the eikonal approximation with the Franck–Condon approximation, where the ion–molecule relative motion is described by rectilinear trajectories, and the molecular nuclei are assumed to remain fixed at their equilibrium positions during the collision.

**Figure 9:**
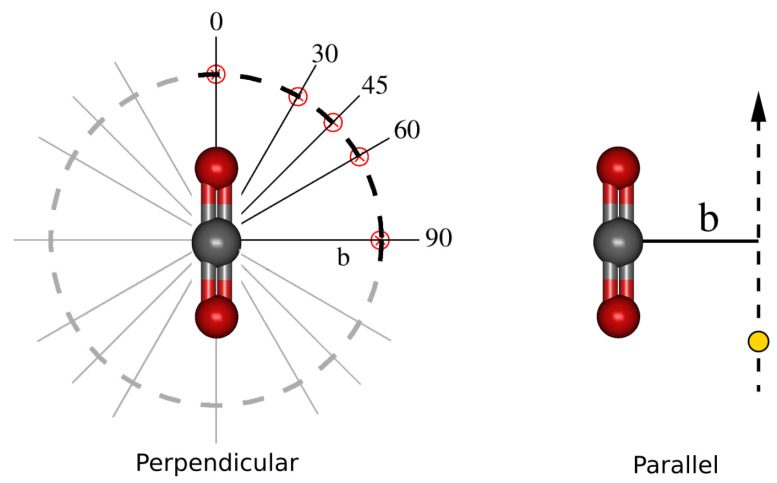
Scheme with the two families of projectile trajectories employed to study H^+^ + CO_2_ collisions: on the left, the t_⊥_ family with v perpendicular to the molecular axis, ρ; on the right, the t_‖_ family, with v parallel to ρ. The t_⊥_ family includes subfamilies with different values of the angle α between the impact parameter vector, ***b***, and ρ being α=0, 30°, 45°, 60°, and 90°.

In order to obtain OAXSs, several trajectory orientations are considered. Each trajectory, R(t), is characterized by the impact parameter b and the velocity v vectors: R(t)=b+vt. The projectile trajectories of a given family share the same $\hat{v}$ and $\hat{b}$, with a set of values for $0\leq b\leq b_{\max}\approx 15\;\mathrm{bohr}$; this last value is such that the electron capture probability is negligible. The trajectory families considered are indicated in Figure 9. For trajectories t_‖_, v is parallel to the molecular axis, ρ, while b is perpendicular to ρ; the target+projectile system preserves the C_*s*_ symmetry along the complete collision. For trajectories t_⊥_, v is perpendicular to ρ, while b can be at any angle α with respect to ρ. We have considered trajectories with α={0,30,45,60,90} degree.

For each trajectory (b,v), the scattering wave function Ψ(r,t) is a solution of the eikonal equation (see [31]):

(9) $$H_{\mathrm{el}}\Psi =i{\left.\frac{\partial \Psi }{\partial t}\right|}_{r}$$

where ***r*** are the electronic coordinates, and $H_{\mathrm{el}}$ the clamped-nuclei electronic Hamiltonian in the Born–Oppenheimer approximation for the H^+^ + CO_2_ system.

The eikonal equation must be solved with the initial condition:

(10) $$\lim_{t\to -\infty }\Psi =\Phi_{k}\left(r\right)D_{k}\left(r,t\right)$$

where $\Phi_{k}$ is the electronic initial wave function. In our calculation, $\Phi_{k}$ is the electronic function of CO_2_($X^{1}\Sigma_{g}^{+}$) at the equilibrium geometry. $D_{k}$ is the plane-wave translation factor that describes the translation of the electrons with respect to the origin (see, e.g., [31]). In our model, Ψ is expressed as a linear combination of molecular wave functions $\chi_{l}\left(r;R\right)$, which are approximate eigenfunctions of $H_{\mathrm{el}}$, calculated along each nuclear trajectory:

(11) $$\Psi \left(r,t\right)=D\left(r,R\right)\sum_{j}c_{j}\left(t\right)\chi_{j}\left(r;R\right)\exp\left[-i\int_{0}^{t}\;\;E_{j}\left(R\right)dt\right],$$

where *D* is a common translation factor (see [32]) that fulfils:

(12) $$\chi_{j}D∼\Phi_{j}D_{j}$$

as R→∞. In the present work, we have employed a common translation factor based on the switching function of Errea et al. [33].

The CT process takes place through transitions between the molecular states driven by the non-adiabatic couplings:

(13) $$M_{ij}=\langle \chi_{i}D\left|H_{\mathrm{el}}-i{\left.\frac{\partial }{\partial t}\right|}_{r}\right|\chi_{j}D\rangle .$$

Defining Z=vt,

(14) $$\frac{\partial }{\partial t}=v\frac{\partial }{\partial Z},$$

and the couplings can be expressed (see [32])

(15) $$M_{ij}=iv\langle \chi_{i}\left|{\left.\frac{\partial }{\partial Z}\right|}_{r}\right|\chi_{j}\rangle +A_{ij},$$

where $A_{ij}$ are the corrections due to the translation factor. In practice, the substitution of the expansion (11) into the eikonal equation yields a set of differential equations for the coefficients $c_{j}$; the solution provides the transition probabilities and cross sections. Explicitly, for a given trajectory (b,v), the transition probability to a different state $\Phi_{l}$ is:

(16) $${\hat{P}}_{kl}\left(b,v\right)=\lim_{t\to \infty }{\left|c_{l}\left(t\right)\right|}^{2},$$

where $\Phi_{k}$ and $\Phi_{l}$ are, respectively, the asymptotic forms of the molecular states $\chi_{k},\chi_{l}$. In our calculations, the entrance channel *k* is the molecular state dissociating into H^+^ + CO_2_($X^{1}\Sigma_{g}^{+}$) and *l* the CT channels. The CT transition probability, ${\hat{P}}_{CT}$, is the sum of the probabilities to all CT channels.

We define the total cross section for a given trajectory orientation and velocity as:

(17) $${\hat{\sigma }}_{kl}\left(\hat{b},v\right)=2\pi \int_{0}^{\infty }\;b{\hat{P}}_{kl}\left(b,v\right)db.$$

The OAXS is:

(18) $$\sigma_{kl}\left(v\right)=\frac{1}{4\pi }\int d\Omega \int db{\hat{P}}_{kl}\left(b,\Omega \right),$$

with Ω the solid angle that defines the orientation of ***v*** with respect to ρ that is fixed during the collision. Using a 6-point integration, and taking advantage of the symmetry of the system, one obtains [17]:

(19) $$\sigma_{kl}\left(v\right)=\int db\left[\frac{1}{3}{\hat{P}}_{kl}^{\|}\left(b\right)+\frac{2}{3}{\hat{P}}_{kl}^{⊥}\left(b\right)\right],$$

where ${\hat{P}}_{kl}^{\|}$ and ${\hat{P}}_{kl}^{⊥}$ are, respectively, the transition probabilities for the trajectory families t_‖_ and t_⊥_ of Figure 9.

The transition probability ${\hat{P}}_{kl}^{\|}$ does not depend on the orientation of ***b***, but one has to consider several orientations of ***b*** of the perpendicular trajectories to calculate the OAXS. This involves the numerical integration over dα of the probabilities ${\hat{P}}_{kl}^{⊥}$, with α the angle between ***b*** and ρ (see Figure 9). A simple four-point rule for the integration in α leads to a formula for the OAXS in terms of the cross sections calculated for three-trajectory (3t) orientations [17]:

(20) $$\begin{array}{c} \sigma_{kl}^{3t}=\frac{2\pi }{3}\left[\int_{0}^{\infty }bdb{\hat{P}}_{kl}^{\|}\left(b\right)+\int_{0}^{\infty }bdb{\hat{P}}_{kl}^{⊥}\left(b,\alpha =0\right)+\int_{0}^{\infty }bdb{\hat{P}}_{kl}^{⊥}\left(b,\alpha =\pi /2\right)\right]=\\ \frac{1}{3}\left[{\hat{\sigma }}_{kl}^{\|}+{\hat{\sigma }}_{kl}^{⊥}\left(\alpha =0\right)+{\hat{\sigma }}_{kl}^{⊥}\left(\alpha =\pi /2\right)\right], \end{array}$$

previously applied in [19,20]. In the present work, we have also considered 8- and 12-point integration rules. We obtain the 4-trajectory (4t) formula:

(21) $$\begin{array}{cc} & \sigma_{kl}^{4t}=\frac{1}{3}{\hat{\sigma }}_{kl}^{\|}+\frac{1}{6}\left[{\hat{\sigma }}_{kl}^{⊥}\left(\alpha =0\right)+2{\hat{\sigma }}_{kl}^{⊥}\left(\alpha =\pi /4\right)+{\hat{\sigma }}_{kl}^{⊥}\left(\alpha =\pi /2\right)\right], \end{array}$$

and the 5-trajectory (5t) formula:

(22) $$\begin{array}{cc} & \sigma_{kl}^{5t}=\frac{1}{3}{\hat{\sigma }}_{kl}^{\|}+\frac{1}{9}\left[{\hat{\sigma }}_{kl}^{⊥}\left(\alpha =0\right)+2{\hat{\sigma }}_{kl}^{⊥}\left(\alpha =\pi /6\right)+2{\hat{\sigma }}_{kl}^{⊥}\left(\alpha =\pi /3\right)+{\hat{\sigma }}_{kl}^{⊥}\left(\alpha =\pi /2\right)\right]. \end{array}$$

We have also applied the 6-trajectory (6t) formula:

(23) $$\begin{array}{c} \sigma_{kl}^{6t}=\frac{1}{3}{\hat{\sigma }}_{kl}^{\|}+\frac{1}{9}\left[{\hat{\sigma }}_{kl}^{⊥}\left(\alpha =0\right)+\frac{3}{2}{\hat{\sigma }}_{kl}^{⊥}\left(\alpha =\pi /6\right)+{\hat{\sigma }}_{kl}^{⊥}\left(\alpha =\pi /4\right)+\frac{3}{2}{\hat{\sigma }}_{kl}^{⊥}\left(\alpha =\pi /3\right)+\right.\\ \left.{\hat{\sigma }}_{kl}^{⊥}\left(\alpha =\pi /2\right)\right] \end{array}$$

As mentioned previously for orientation-dependent probabilities and cross sections, the sum of orientation-averaged probabilities and cross sections for CT channels yields the corresponding orientation-averaged quantities $P_{\mathrm{CT}}$, $\sigma_{\mathrm{CT}}$.

### 3.2. Molecular Model

Our model aims at describing, using the multi-reference configuration interaction method (MRCI), the first five singlet electronic states of the (HCO_2_)^+^ system, which correlate to three asymptotic channels: (i) the entrance channel is H^+^ + CO_2_($X^{1}\Sigma_{g}^{+}$); (ii) the lowest CT channel is doubly degenerate and produced when the proton captures one electron into its 1s orbital from the HOMO ($\pi_{g}$) molecular orbital of CO_2_, leading to H(1s) + CO_2_^+^($X^{2}\Pi_{g}$); and (iii) a second, also doubly degenerate, CT channel in which one electron is removed from the HOMO-1 ($\pi_{u}$) CO_2_ orbital and leads to H(1s) + CO_2_^+^($A^{2}\Pi_{u}$). Our model uses a basis set of Widmark et al. [34]: the 3s2p1d for carbon and oxygen and 2s1p for hydrogen. Electronic wave functions are obtained with the MOLPRO [35] MRCI program, with the molecular orbitals (MOs) obtained in a complete active space-self-consistent field (CASSCF) calculation. Depending on the relative orientation of the projectile trajectory with respect to the molecular axis, the target+projectile system runs through configurations of C_*s*_ or C_1_ symmetries. For C_*s*_ trajectories, eight electrons are distributed into 5 a^′^ and 2 a^″^ orbitals, while keeping the first 7 a^′^ orbitals always doubly occupied; the entrance channel is of A’ symmetry and, consequently, only three electronic states need to be considered in the CASSCF, with equal weights. For C_1_ trajectories, the eight active electrons are distributed into seven orbitals; five states are obtained in the CASSCF, with equal weights. The CASSCF MOs and reference configurations are used in the MRCI calculations to obtain the wave functions by allowing all single and double excitations from the reference configurations into the virtual orbitals.

## 4. Conclusions

We have carried out the first ab initio calculation of the electron capture process between proton and CO_2_, employing an expansion in terms of molecular functions of the HCO_2_^+^ system. The large experimental cross sections at relatively low collision energies can be attributed to the quasi-degeneracy of the entrance channel, H^+^ + CO_2_($X^{1}\Sigma_{g}^{+}$), and the main capture channel H + CO_2_^+^($X^{2}\Pi_{g}$). This quasi-degeneracy underscores the critical importance of quantum chemical precision at very low energies. Consequently, the first objective of our study was to assess the impact of this effect on dynamical results through two-state model calculations. In this model, two diabatic states are coupled by an exponential interaction term, and the energies account for long-range interactions. We have demonstrated that this simplified model effectively captures the essential physical mechanisms underlying the electron transfer process. A comparison of the two-state calculations reveals that swapping the asymptotic energies introduces cross-section variations comparable to experimental uncertainties for collision energies below 0.8 keV/u, while such variations become negligible at higher energies. These findings validate our molecular expansion approach using MRCI wave functions and provide robust support for its accuracy in describing the dynamics of the system.

Molecular expansions are generally well-suited for studying ion–atom/molecule collisions at low energies. However, a significant practical challenge inherent to these expansions is the indeterminacy of the sign of the molecular wave functions. Typically, the sign is chosen to ensure smooth couplings. However, in the H^+^ + CO_2_ system, we have found that this criterion is not always appropriate. Specifically, for trajectories where the plane Z=0 is a symmetry plane of the CO_2_ molecule, the smooth evolution of the molecular wave functions results in a sudden sign flip in some couplings at the point of closest approach of the trajectory. This behavior is likely to occur in other systems as well, particularly when certain electronic states exhibit antisymmetry under reflection in the Z=0 plane. Such cases arise when the dominant electronic configuration involves singly occupied molecular orbitals that are antisymmetric with respect to reflection in this plane.

To compare with the available experimental results, it is necessary to compute orientation-averaged cross sections. Our method, previously applied to collisions with H_2_, involves solving the dynamical problem for a limited set of trajectory orientations. Although CO_2_ is a linear molecule, we have found that the symmetry of its HOMO significantly reduces the cross section for trajectories where both the impact parameter and the velocity vector are perpendicular to the internuclear axis of CO_2_. In our earlier works, this particular family of trajectories was assigned a weight of 1/3 in the averaging process, introducing a notable bias in the present calculation. By extending the analysis to include additional trajectory orientations, we have achieved convergence in the averaged cross section, which shows remarkable agreement with the experimental data.

## Figures and Tables

**Table 1:** Electronic energy differences (in eV) at b=8 bohr and R=17 bohr of (H + CO_2_)^+^ for different trajectories, and comparison with experimental data.

Channel	t_‖_^a^	t_⊥_^b^	t_⊥_^c^	Exp
H^+^ + CO_2_($X^{1}\Sigma_{g}^{+}$)	0	0	0	0
H + CO_2_^+^($X^{2}\Pi_{g}$)	−0.094	−0.20	−0.10	0.172 ^d^
H + CO_2_^+^($A^{2}\Pi_{u}$)	4.0	3.7	3.99	3.71 ^e^

^a^ t_‖_, C_*s*_, A’, $3.3\times 10^{5}$ contracted CSFs. ^b^ ${t_{⊥}}_{30,45,60}$, C_1_, A, $8.0\times 10^{5}$ contracted CSFs. ^c^ ${t_{⊥}}_{0,90}$, C_s_, A’, $3.3\times 10^{5}$ contracted CSFs. ^d^ [24]. ^e^ [25].

**Figure A1:**
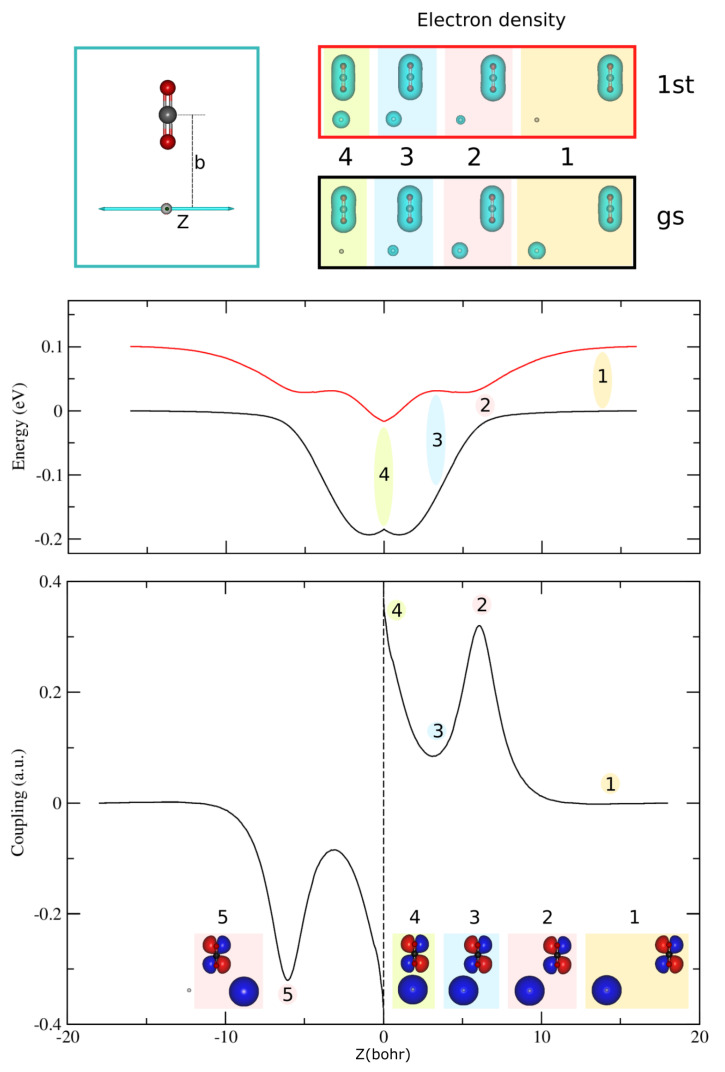
Potential energy curves (middle diagram) of the first two electronic states of (H-CO_2_)^+^ along a projectile trajectory perpendicular to the CO_2_ internuclear axis (top left) with impact parameter b=8 bohr, and the non-adiabatic coupling between the two states (bottom) with the molecular orbitals depicted at five stages of the collisions, drawn in color squares and marked next to the energies and coupling lines at approximately their corresponding value of *Z*. At the first four stages, the electronic density for the two electronic states is shown in the top-right diagram. The two molecular orbitals depicted resemble a $\pi_{g}$ on the CO_2_ molecule and the 1s on the projectile.

## Data Availability

The original data presented in the study are openly available at e-cienciaDatos https://doi.org/10.21950/LIQ81K.

## Abbreviations

The following abbreviations are used in this manuscript:

CT	Charge transfer
OAXS	Orientation-averaged cross section
HOMO	Highly occupied molecular orbital
MRCI	Multi-reference configuration interaction
CASSCF	Complete active space self-consistent field
CSF	Configuration state function
MO	Molecular orbital

## Appendix A. Sign-Flip

In this appendix, we discuss the sudden sign change of the dynamical coupling $M_{12}$ along the trajectory and t_⊥0_ with $\hat{b}$ along the OCO axis, as shown in the first panel of Figure A1, but the same arguments explain the sign flip of this coupling along the trajectory t_⊥_. The electronic energies and the coupling $M_{12}$ are also presented in Figure A1 for a $t_{⊥0}$ trajectory with b=8.0 bohr.

The wave functions of the ground and the first excited electronic states of (H-CO_2_)^+^ are dominated by two configurations $\left|\right|\Phi_{c}\pi_{g}^{2}\left|\right|$ and $\left|\right|\Phi_{c}\pi_{g}\phi_{1s}\left|\right|$, where $\Phi_{c}$ represent the doubly occupied first ten MOs:

(A1) $$\begin{array}{ccc} \psi_{\mathrm{gs}} & = & c_{11}\left|\right|\Phi_{c}\pi_{g}^{2}\left|\right|+c_{12}\left|\right|\Phi_{c}\pi_{g}\phi_{1s}\left|\right|+\ldots \end{array}$$

(A2) $$\begin{array}{ccc} \psi_{1\mathrm{st}} & = & c_{21}\left|\right|\Phi_{c}\pi_{g}^{2}\left|\right|+c_{22}\left|\right|\Phi_{c}\pi_{g}\phi_{1s}\left|\right|+\ldots \end{array}$$

We have checked that the MOs do not significantly change along the trajectory, as shown in Figure A1, and, in particular, we do not find sign flips of these MOs. In the limit Z→∞ we have:

(A3) $$\begin{array}{ccc} \psi_{\mathrm{gs}} & ∼ & \left|\right|\Phi_{c}\pi_{g}\phi_{1s}\left|\right|\\ \psi_{1\mathrm{st}} & ∼ & \left|\right|\Phi_{c}\pi_{g}^{2}\left|\right|, \end{array}$$

In agreement with the densities plotted in Figure A1.

For $Z\to 0^{+}$, the coefficients $c_{ij}$ of the main configurations have the form

(A4) $$\left(\begin{array}{cc} c_{11} & c_{12}\\ c_{21} & c_{22} \end{array}\right)\equiv \left(\begin{array}{cc} f(Z) & g(Z)\\ g(Z) & -f(Z) \end{array}\right)$$

and we have found that these coefficients can be fitted to the expressions:

(A5) $$\begin{array}{ccc} f(Z) & \approx & A-CZ^{2} \end{array}$$

(A6) $$\begin{array}{ccc} g(Z) & \approx & BZ \end{array}$$

with A=0.93, B=0.325 for the case illustrated in Figure A1. In order to set the sign of the wave functions for Z<0, one has to take into account that the electronic wave functions must be identical at $Z=\pm Z_{0}$, and since the signs of the MOs do not change at Z=0, the signs of coefficients of the corresponding configurations must change. For small $Z_{0}$, this can be obtained by taking $\psi_{\;1\mathrm{st}}\left(-Z_{0}\right)=-\psi_{1\mathrm{st}}\left(Z_{0}\right)$. Accordingly, we write:

(A7) $$\left(\begin{array}{cc} c_{11} & c_{12}\\ c_{21} & c_{22} \end{array}\right)\equiv \left(\begin{array}{cc} f(Z) & g(Z)\\ g(Z)\mathrm{sgn}(Z) & -f(Z)\mathrm{sgn}(Z) \end{array}\right).$$

Neglecting the translation factor corrections, the ensuing coupling between the two functions is:

(A8) $$M_{12}=\langle \psi_{1}|d\psi_{2}/dZ\rangle =\left(\begin{array}{cc} c_{11} & c_{12} \end{array}\right)\left(\begin{array}{c} dc_{21}/dZ\\ dc_{22}/dZ \end{array}\right)$$

Operating,

(A9) $$\begin{array}{ccc} \frac{dc_{21}}{dZ} & = & \frac{dg(Z)}{dZ}\mathrm{sgn}\left(Z\right)+g\left(Z\right)\frac{d\mathrm{sgn}(Z)}{dZ} \end{array}$$

(A10) $$\begin{array}{ccc} \frac{dc_{22}}{dZ} & = & -\frac{df(Z)}{dZ}\mathrm{sgn}\left(Z\right)-f\left(Z\right)\frac{d\mathrm{sgn}(Z)}{dZ} \end{array}$$

then

(A11) $$M_{12}\left(Z\right)=B\left(A+CZ^{2}\right)\mathrm{sgn}\left(Z\right),$$

which does not include the derivative of the sgn function. The fitting of the CI coefficients leads to AB≈0.302 bohr^−1^, in good agreement with the ab initio value at Z=0 (Figure A1), which supports our interpretation of the coupling in terms of two configurations for Z≈0. In conclusion, given that the $\pi_{g}$ MO is antisymmetric upon reflection on the plane at Z=0, the smooth evolution of the wave functions is obtained by enforcing a sign change of the function $\phi_{1\mathrm{st}}$ at Z=0, which leads to the sign flips of some dynamical couplings.
